# Supplementary material for: Abasy Atlas: a comprehensive inventory of systems, global network properties and systems-level elements across bacteria
Source: Database (Oxford). 2016 May 30;2016:baw089. doi: 10.1093/database/baw089 (PMC4885605; doi:10.1093/database/baw089)
Supplement: Supplementary Data [file supp_2016_baw089_index.html]

Abasy Atlas: a comprehensive inventory of systems, global network properties and systems-level elements across bacteria — Supplementary Data 

# Abasy Atlas: a comprehensive inventory of systems, global network properties and systems-level elements across bacteria

## Supplementary Data

files

- Supplementary Data - zip file
