# Supplementary figures and images for: Abasy Atlas: a comprehensive inventory of systems, global network properties and systems-level elements across bacteria
Source: Database (Oxford). 2016 May 30;2016:baw089. doi: 10.1093/database/baw089 (PMC4885605; doi:10.1093/database/baw089)

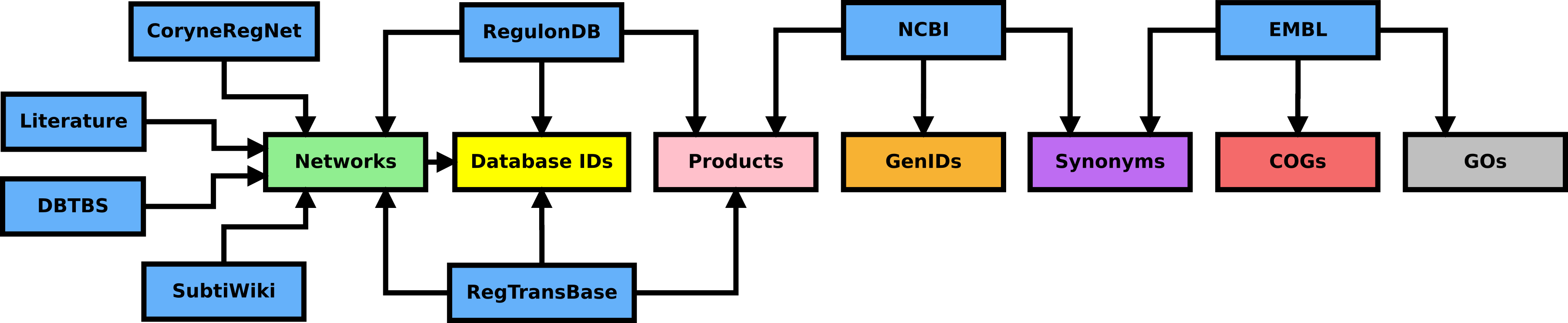

Supplement: Supplementary Data [file supp_baw089_suppl_data.zip › DataSources-wbk.png]

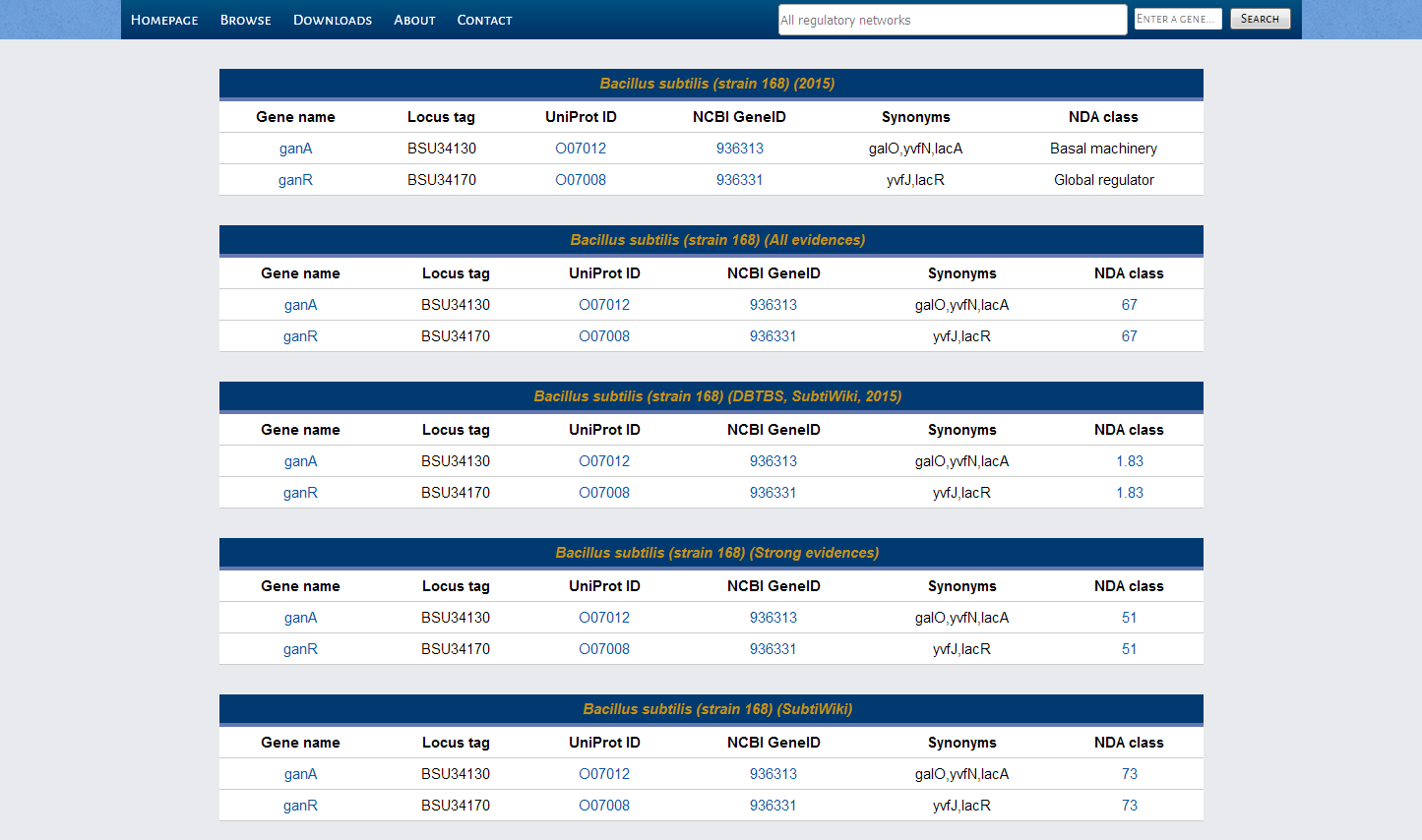

Supplement: Supplementary Data [file supp_baw089_suppl_data.zip › Query-wbk.png]

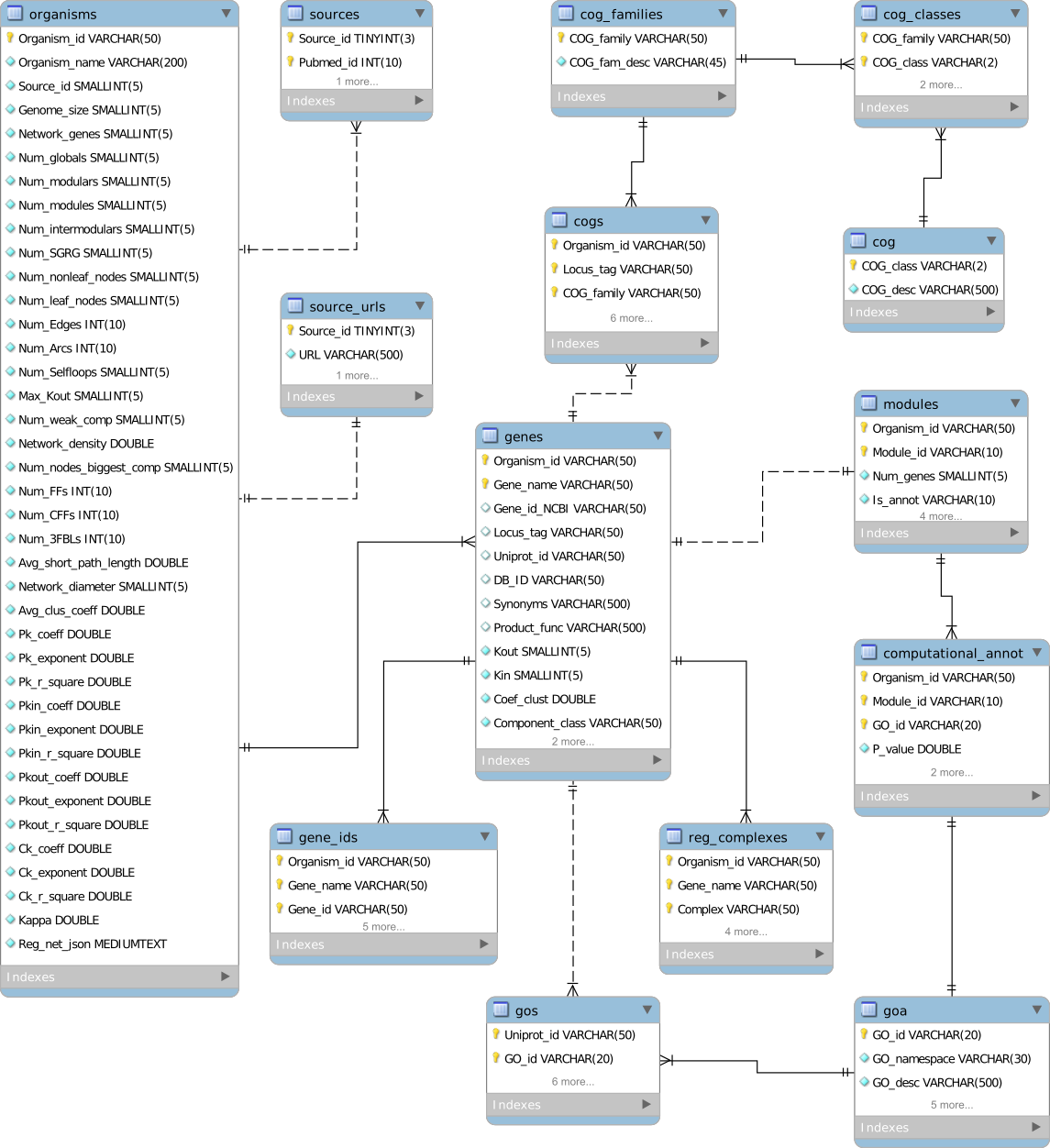

Supplement: Supplementary Data [file supp_baw089_suppl_data.zip › BactSytsDB-ER-crop-wbk.png]
